# Decoding information about cognitive health from the brainwaves of sleep

**DOI:** 10.1038/s41598-023-37128-7

**Published:** 2023-07-15

**Authors:** Noor Adra, Lisa W. Dümmer, Luis Paixao, Ryan A. Tesh, Haoqi Sun, Wolfgang Ganglberger, Mike Westmeijer, Madalena Da Silva Cardoso, Anagha Kumar, Elissa Ye, Jonathan Henry, Sydney S. Cash, Erin Kitchener, Catherine L. Leveroni, Rhoda Au, Jonathan Rosand, Joel Salinas, Alice D. Lam, Robert J. Thomas, M. Brandon Westover

**Affiliations:** 1 Department of Neurology, Massachusetts General Hospital (MGH), Boston, MA USA; 2 Clinical Data Animation Center (CDAC), MGH, Boston, MA USA; 3 Henry and Allison McCance Center for Brain Health, Massachusetts General Hospital (MGH), 55 Fruit Street, Boston, MA 02114 USA; 4 University of Groningen, Groningen, The Netherlands; 5 Department of Neurology, Washington University School of Medicine in St. Louis, St. Louis, MO USA; 6 Sleep and Health Zurich, University of Zurich, Zurich, Switzerland; 7 Utrecht University, Utrecht, The Netherlands; 8 Boston University Chobanian and Avedisian School of Medicine, Boston, MA USA; 9 New York University Grossman School of Medicine, New York, NY USA; 10 Division of Pulmonary, Critical Care, and Sleep, Department of Medicine, Beth Israel Deaconess Medical Center (BIDMC), Boston, MA USA

**Keywords:** Cognitive neuroscience, Neurology

## Abstract

Sleep electroencephalogram (EEG) signals likely encode brain health information that may identify individuals at high risk for age-related brain diseases. Here, we evaluate the correlation of a previously proposed brain age biomarker, the “brain age index” (BAI), with cognitive test scores and use machine learning to develop and validate a series of new sleep EEG-based indices, termed “sleep cognitive indices” (SCIs), that are directly optimized to correlate with specific cognitive scores. Three overarching cognitive processes were examined: total, fluid (a measure of cognitive processes involved in reasoning-based problem solving and susceptible to aging and neuropathology), and crystallized cognition (a measure of cognitive processes involved in applying acquired knowledge toward problem-solving). We show that SCI decoded information about total cognition (Pearson’s r = 0.37) and fluid cognition (Pearson’s r = 0.56), while BAI correlated only with crystallized cognition (Pearson’s r = − 0.25). Overall, these sleep EEG-derived biomarkers may provide accessible and clinically meaningful indicators of neurocognitive health.

## Introduction

Our society is aging at unprecedented rates, with the average human lifespan increasing globally. Aging-associated cognitive decline impairs daily functioning^1^, increases the frequency of hospitalization and emergency visits^2^, and is associated with heightened risk of multimorbidity^3,4^. Patients with cognitive impairment and dementia also face barriers to diagnosis^5,6^, leading to delayed detection that further hinders appropriate care, treatment, and patient functioning. Developing easily repeatable biomarkers of “brain health” (i.e., the combined preservation of optimal brain integrity and cognitive function) could facilitate early detection of current or future cognitive impairment risk which may in turn guide potential therapeutic opportunities for patients.

Sleep-physiology based metrics are attractive as indicators of brain health because changes in sleep architecture are not only strongly associated with aging, but also with cognitive decline and a wide range of neuropathologic changes, suggesting that sleep may provide a general-purpose window into brain health. For example, people advancing into the fifth decade of age present with increased sleep-onset latency and sleep fragmentation and decreased total sleep time and sleep efficiency^7,8^. They also exhibit reductions in slow-wave sleep (SWS) percent, rapid eye movement (REM) percent, and density, duration and amplitude of sleep spindles, and increases in non-REM stage 1 (N1) percent and nighttime wakefulness^8–10^. These age-related sleep changes have been linked to subsequent cognitive decline^11^ and increased risk of dementia^12,13^.

One recently proposed sleep EEG-based biomarker of brain health is “brain age” (BA)^14^. Its difference from chronological age, called the “brain age index” (BAI), characterizes the extent to which an individual’s observed neurophysiologic functioning during sleep deviates from what would be expected for their chronological age. In excess, BAI has been linked to higher mortality^15^ and an underlying burden of disease, including dementia^16^, HIV infection^17^, hypertension^14^, and diabetes^14^. Although BAI gives insight into the general functional capacity of the brain, it is not explicitly designed to decode information about neuroanatomic integrity and its relationship with cognition has not yet been evaluated.

Here, our aim was to take a novel approach to measuring brain health by developing methods to decode neurocognitive information from sleep. Specifically, we developed a series of novel markers of brain health termed Sleep Cognitive Indices (SCIs). Unlike BAI, the SCIs are explicitly designed to correlate with specific components of cognition. Such indicators of brain health could be important for identifying age-related brain diseases which preferentially affect specific aspects of cognition, or for tracking the effects of interventions targeted at specific cognitive domains. We hypothesized that specific combinations of sleep-EEG features would be correlated with performance on specific cognitive tasks and that it may thus be possible to develop EEG-based indicators specifically correlated with different types of cognitive abilities. In comparison, we expected participants with elevated BAI to perform worse on cognitive assessments but reasoned that this correlation is likely nonspecific since BAI was developed to predict age.

## Methods

### Design and participants

We conducted a single-center, cross-sectional observational study consisting of adults (≥ 18 years of age) who underwent diagnostic polysomnography (PSG) between November 2018 and October 2019 at the Massachusetts General Hospital Sleep Laboratory. Enrolled participants completed a cognitive test battery within 40 days of their PSG. Patients were excluded if they had a baseline diagnosis of dementia or a learning disability, were unable to perform the cognitive tests due to a lack of English proficiency or impairment (motor, visual, or hearing), or if they had prior experience with the cognitive test battery. This study of human subjects was approved by the Mass General Brigham Institutional Review Board. All methods were performed in accordance with the study protocol and the Declaration of Helsinki. Written informed consent was provided by all participants. The number of subjects and their characteristics are summarized in Table 1.

**Table 1:** Baseline characteristics of study patients (N = 150).

	Female (N = 84)	Male (N = 66)
Age, years, mean ± SD	47.0 ± 17.2	51.0 ± 18.3
Years of education, mean ± SD	16.1 ± 2.8	16.9 ± 3.2
Race		
White, n (%)	61 (72.6%)	56 (84.8%)
Black, n (%)	6 (7.1%)	1 (1.5%)
Hispanic/Latino, n (%)	1 (1.2%)	0 (0.0%)
Asian, n (%)	5 (6.0%)	4 (6.1%)
Multiracial, n (%)	11 (13.1%)	5 (7.6%)
Hollingshead index, median [IQR]	48.0 [38.0, 58.0]	56.0 [47.5, 63.8]
Employed or self-employed, n (%)	48 (57.2%)	34 (51.5%)
Marital status (married), n (%)	36 (42.9%)	32 (48.5%)
Current smoker, n (%)	2 (2.4%)	4 (6.1%)
AUDIT, median [IQR]	2.0 [1, 3]	2.0 [0, 4]
History of alcohol abuse, n (%)	3 (3.6%)	11 (16.7%)
History of substance abuse, n (%)	6 (7.1%)	12 (18.2%)
Body mass index, kg/m^2^, median [IQR]	28.5 [23.9, 33.5]	28.4 [25.4, 32.5]
Charlson Comorbidity Index, median [IQR]	1.0 [0.0, 3.0]	1.0 [0.0 ,3.0]
PHQ-4, median [IQR]	2.0 [1.0, 4.0]	2.0 [0.0, 6.0]
Mediterranean Diet Score, median [IQR]	6.0 [4.0, 8.0]	5.0 [4.0, 7.0]
Family history of dementia, n (%)*	37 (44.0%)	23 (34.8%)
Family history of Parkinson’s disease, n (%)*	15 (17.9%)	7 (10.6%)
PSQI, median [IQR]	8.00 [5.3, 12.0]	8.00 [4.8, 12.0]
ESS, median [IQR]	10.0 [5.0, 13.0]	9.0 [4.0, 12.0]
AHI, median [IQR]	2.2 [0.5, 5.9]	6.2 [2.0, 13.9]
Normal (< 5), n (%)	60 (71.4%)	26 (39.4%)
Mild sleep apnea (5 ≤ AHI < 15), n (%)	21 (25.0%)	24 (36.4%)
Moderate sleep apnea (15 ≤ AHI < 30), n (%)	3 (3.6%)	11 (16.7%)
Severe sleep apnea (AHI ≥ 30), n (%)	0 (0.0%)	5 (7.6%)
Total Sleep Time, median [IQR]	6.54 [5.8, 7.3]	6.21 [5.0, 6.9]
Sleep Efficiency, median [IQR]	0.85 [0.7, 0.9]	0.82 [0.7, 0.9]
REM Latency, median [IQR]	2.13 [2.4, 3.8]	2.66 [1.8, 4.3]
%N1, median [IQR]	6.74 [3.6, 9.6]	8.71 [5.2, 13.9]
%N2, median [IQR]	60.63 [51.4, 67.7]	63.50 [52.9, 69.4]
%N3, median [IQR]	16.31 [10.7, 22.2]	12.33 [4.1, 20.2]
%R, median [IQR]	16.12 [9.9, 20.1]	14.79 [10.1, 18.2]
WASO, median [IQR]	8.01 [3.5, 15.2]	12.34 [4.1, 23.1]
PSG referral reasons		
Sleep apnea evaluation, n (%)	42 (50.0%)	43 (65.2%)
Sleepiness, n (%)	42 (50.0%)	33 (50.0%)
Snoring, n (%)	33 (39.3%)	30 (45.5%)
Insomnia, n (%)	26 (31.0%)	21 (31.8%)
Restless legs syndrome, n (%)	14 (16.7%)	16 (24.2%)
Chronic fatigue, n (%)	2 (2.4%)	1 (1.5%)
REM sleep behavior disorder, n (%)	1 (1.2%)	1 (1.5%)
Narcolepsy, n (%)	1 (1.2%)	1 (1.5%)
Research, n (%)	0 (0.0%)	1 (1.5%)
Non-restorative sleep, n (%)	1 (1.2%)	0 (0.0%)
Idiopathic hypersomnia, n (%)	1 (1.2%)	0 (0.0%)

* Family history of one patient was unknown as patient was adopted.

*Abbreviations* SD, Standard deviation; IQR, Interquartile range; AUDIT, Alcohol use disorders identification test; PHQ-4, Patient health questionnaire for depression and anxiety—4 item; PSQI, Pittsburgh Sleep Quality Index; ESS, Epworth Sleepiness Scale; AHI, apnea–hypopnea index (# apnea events per hour of sleep) at 4% desaturation for hypopnea.

### Sleep signal preprocessing

Electroencephalogram (EEG) signals were recorded from six scalp electrodes: frontal (F3, F4), central (C3, C4), and occipital (O1, O2), each referenced to the contralateral mastoid (M1, M2). EEG signals were recorded at 512 Hz and downsampled to 200 Hz before analysis. These signals were then band-pass filtered between 0.1 and 20 Hz and noncerebral artifacts were removed using a previously described filtering method^18^.

The American Academy of Sleep Medicine (AASM) provides guidelines for classifying consecutive 30-s epochs of EEG signals into 5 “stages”^19^, including awake (W), rapid eye movement (REM) sleep, and 3 stages of non-REM sleep (N1, N2, N3). EEG epochs were classified following these AASM guidelines by licensed sleep technicians and the assigned stages were subsequently reviewed and revised as needed by a sleep physician. Only central electrode signals (C3-M2 and C4-M1) were used for our main analysis, as the public sleep dataset that we used for external validation, the Sleep Heart Health Study included only central electrodes. We further explored model performance when either occipital or frontal electrodes were available for analysis in addition to central electrodes.

### Spindle and slow oscillation characterization

Sleep spindle and slow oscillation features were obtained using Luna software^9^ (http://zzz.bwh.harvard.edu/luna/). Spindle detections were included only for epochs scored as N2 and N3. A single electrocardiogram (ECG) electrode was zero-phase band-pass filtered from 0.3 to 40 Hz and used to apply ECG-correction to remove ECG artifacts from the EEG signals. Slow oscillations were detected by band-pass filtering between 0.2 and 4.5 Hz. Positive-to-negative zero-crossings were then detected in the filtered signal, and intervals between 0.8 and 2-s were designated as slow oscillations if they had a negative peak higher than the median across all zero-crossings and a peak-to-peak amplitude higher than the median. All spindle and slow oscillation features used for analysis are summarized in Table 2.

**Table 2:** Sleep features included in the Sleep Cognitive Index (SCI) model (BAI used a subset of features used in SCI, i.e., the last two feature domains).

Domain	Feature	# using central electrode^^^	# using electrodes from three regions
Macro-structure	TST: total sleep time (N1, N2, N3, and REM) in minutes	1	
	WASO: wake after sleep onset in minutes	1	
	SE: sleep efficiency, time of sleep divided by TST	1	
	TBB: total time in bed, time of sleep and awake	1	
	SL: latency from lights off to the first epoch of sleep in minutes	1	
	RL: latency from lights off to the first epoch of R in minutes	1	
	Perc_X: % of time spent in each sleep stage	4	
Spindle (N2 and N3)	AMP: mean spindle amplitude (uV or mV units)	2	
	DENS: spindle density (count per minute)	2	
	DUR: mean spindle duration (core + flanking region)	2	
	FFT: mean spindle frequency	2	
	FWHM: mean spindle full width at half maximum	2	
	NOSC: mean number of oscillations per spindle	2	
	SYMM2: mean spindle folded-symmetry metric	2	
Spindle-SO coupling (N2 and N3)	COUPL_ANGLE: circular mean of SO phase at spindle peak	2	
	COUPL_MAG: intra-trial phase clustering metric (ITPC)	2	
	COUPL_OVERLAP: # of spindles overlapping a SO	2	
	COUPL_PV: asymptotic *p*-value for the ITPC statistic	2	
Slow oscillations (SO) (N2 and N3)	SO: # of SO detected	2	
	SO_AMP: median amplitude (of negative peak)	2	
	SO_DUR: median SO duration	2	
	SO_NEG_DUR: median negative peak duration	2	
	SO_POS_DUR: median positive peak duration	2	
	SO_RATE: # SO per minute	2	
	Median SO slope: SO_SLOPE_NEG1: + to − zero-crossing to negative peak SO_SLOPE_NEG2: negative peak to − to + zero-crossing SO_SLOPE_POS1: − to + zero-crossing to positive peak SO_SLOPE_POS2: positive peak to + to − zero-crossing	2 × 4	
Waveform for each stage*	Line length, an integrated measure of signal waveform amplitude and frequency	2 × 5	2 × 5 × 3
	Kurtosis, heavy-tailness of the distribution of the waveform	2 × 5	2 × 5 × 3
Frequency for each stage*	Relative delta / theta / alpha band power, each band has 95% percentile, min, mean, standard deviation across 2-s subepochs within a 30-s epoch	4 × 3 × 5	4 × 3 × 5 × 3
	Delta-to-theta/delta-to-alpha/theta-to-alpha power ratio, each ratio has 95% percentile, min, mean, standard deviation across 2-s subepochs within a 30-s epoch	4 × 3 × 5	4 × 3 × 5 × 3
	Kurtosis of delta / theta / alpha /sigma band spectrogram	4 × 5	4 × 5 × 3
Total number of features		212	532

*Each feature is averaged across all 30-s epochs in a specific sleep stage to represent a whole night, then concatenated over all the sleep stages to represent a whole night; this is where × 5 comes from.

^For the main model, sleep features were extracted from sleep EEG central channels only.

### Sleep macrostructure features

Sleep macrostructure measures were calculated following AASM definitions, including total sleep time (TST), wake after sleep onset (WASO), sleep efficiency (SE), total time in bed (TTB), sleep latency (Sleep_L), and REM latency (REM_L). Percentages of TST spent in N1, N2, N3, and REM were calculated using custom code written in Python (https://www.python.org/). All sleep macrostructure features are summarized in Table 2.

### Cognitive test battery

All participants were asked to complete the NIH Toolbox Cognition Battery^20^. The NIH Toolbox Cognition Battery is one of the core domains in the NIH Toolbox for Assessment of Neurological and Behavioral Function. It consists of seven instruments that assess the following functional constructs: Flanker Inhibitory Control and Visual Attention (ICA), Dimensional Change Card Sort (DCCS; measures cognitive flexibility), List Sorting Working Memory (LSWM), Picture Sequence Memory (PSM; measures visual episodic memory), Pattern Comparison Processing Speed (PCPS), Picture Vocabulary (PV; measures vocabulary comprehension), and Oral Reading Recognition (ORR; measures reading decoding). Of these seven instruments, PV and ORR are classified as measures of crystallized cognition and the rest as measures of fluid cognition. Fluid cognition reflects a collection of cognitive processes involved in problem-solving, abstract thinking, and reasoning that are independent of past knowledge acquired through experience and education. In contrast, crystallized cognition represents a group of cognitive processes that apply prior knowledge from experience and education to solve problems. Although different, these two cognition types are tightly correlated components of total cognition. Despite this association, studies often examine these subdivisions of total cognition separately to better understand and treat neurologic conditions^21,22^. Additionally, both crystallized and fluid cognition have been shown to change with age^23,24^. For detailed instrument information, see Table S1. In addition to scores for individual tests, three composite scores for fluid, crystallized, and total cognition are provided. Absolute scores for each of the seven tests and the three composite scores were used for analyses (all non-age adjusted).

### Statistical analyses

#### Developing the sleep cognitive indices

To develop SCI for specific cognitive measures, we created a series of regression models. The dependent variable of each model was a task’s absolute scores. Independent variables in SCI models included EEG features that were derived from spindles and slow waves, as well as the features in Table 2. Demographic variables, such as age and sex, were not included since our primary aim was to develop EEG-based indicators of neurocognitive health and evaluate how well brain signals alone could capture neurocognitive status, rather than produce accurate predictions of cognitive performance per se. EEG-based models were evaluated with a goodness of fit test (see below) in comparison to a full model with demographic variables. Because the number of independent variables (160 × 3 + 42 + 10 = 532 with all electrodes, 160 + 42 + 10 = 212 with one electrode set, Table 2) exceeded the number of participants (150) in our dataset, we used linear regression with Elastic Net regularization to prevent overfitting and to force regression models to select only the features most relevant to the target task. Note that Elastic Net regularization automatically selects which features to retain in the model, and thus the number of features selected varies depending on the specific prediction task and data used to develop the model. To avoid overestimation of regression performance, model training and feature selection were restricted to training data, while model performance was evaluated strictly on held-out test data. In summary, each SCI model is generated by extracting EEG measures of interest (determined by Elastic Net regularization), multiplying these EEG measures by the regression coefficients of the model of interest (fluid, crystallized, total), and adding the results to obtain a single number (SCI score).

For SCI model optimization and testing model performance, we used nested tenfold cross-validation (CV) (Fig. S1). For each functional construct and cognitive composite score, the outer CV loop separated data into ten folds, where each fold contained 15 distinct participants. Nine folds were used for model fitting (n = 135) and the other fold for model testing (n = 15). This was done ten times, such that testing was performed once on each fold. During model fitting, Elastic Net regression was performed to select the best subset of features and their coefficients. Strict separation of training and test set was maintained to achieve statistically unbiased estimates of out-of-sample performance. Our reported performance results are based on test data only.

In addition to the new SCI models, we calculated the Brain Age Index (BAI) using a previously described machine learning model^14^. BAI includes features from the waveform time domain (e.g. line length and kurtosis which reflect EEG signals complexity) and from the frequency domain (e.g. spectral power of the delta (0.5–4 Hz), theta (4–8 Hz), alpha (8–12 Hz) bands, and their ratios^14,25^). All features are summarized in Table 2. Features of missing sleep stages were imputed using the K-nearest neighbor approach (K = 10). We used Pearson correlations to measure the degree to which the BAI correlates with cognitive test scores. Statistical significance was defined using a *p*-value < 0.05.

Pearson correlation was calculated between cognitive scores and the various SCI and BAI. To compare pairs of correlation coefficients (e.g. to evaluate the difference between the strength of correlations of BAI vs. SCI with each cognitive test), we completed Fisher r-to-z transformations for each pair of correlation coefficients^26^.

To evaluate how well SCI and BAI distinguish individuals who score low versus high on different cognitive tests, we divided participants into three groups of equal size for each cognitive test (1/3 low score, 1/3 medium score, 1/3 high score). We then performed group-level analysis of discriminability using Cuzick’s non-parametric test for trend to examine the statistical significance of the difference in SCI across the different score groups. For individual-level analysis of discriminability, we calculated Receiver Operating Characteristic (ROC) curves and the Area Under the ROC Curve (AUC) for each SCI model. When performing ROC analysis, the medium score group was excluded from this analysis to ensure distinctness between groups.

#### Evaluating cognitive variation related to age, sex, and education

Performance on cognitive tasks in the NIH Toolbox Cognition Battery depends on age^20^. We reasoned that, if our SCI indicators are valid, they should account for age-related variation in cognitive performance. If so, regression models that include age and SCI should explain no more of the variance in cognitive test performance than regression models that include SCI alone. Similar reasoning applies to other biological variables that might correlate with cognitive performance, including years of education and sex. To address these questions, we created a series of nested regression models and compared each submodel using a likelihood ratio test. Specifically, we first fitted two Elastic Net models for each cognitive test: (1) a submodel with EEG features alone (SCI model), and (2) a full model with EEG features, age, years of education, and sex. We then compared models by calculating the log-likelihood of each model and performing a likelihood ratio test to measure the change in deviance for the submodel.

#### External validation

External validation was performed using EEG data from the Sleep Heart Health Study^27–30^ (SHHS), a composite cohort overlapping with the Framingham Heart Study^31^ (FHS). Participants were included if they completed a neuropsychological test battery^32^ in the FHS within 3 years of their SHHS polysomnography exam date. Scores from the following tests were used for the Wechsler Memory Scale (WMS) score calculation: Logical memory—Immediate Recall, Delayed Recall, Recognition; Visual reproductions—Immediate recall, Delayed Recall, Recognition; Paired Associate Learning—Immediate Recall, Delayed Recall. Of the 476 participants in the validation dataset; 152 were subsequently excluded due to incomplete WMS data, with the remaining 324 available for analysis. WMS does not include tests that are directly comparable with the three NIH toolbox composite scores (total, fluid, and crystallized); therefore we correlated the WMS score with all three composite SCI models (total, fluid, crystallized), with the expectation that these constructs are correlated and thus, if the SCI models capture valid physiologic information related to brain health, they should exhibit some measurable (if nonspecific) correlation with WMS scores.

A subset of participants in the FHS cohort was flagged for possible dementia using criteria as previously described^33–36^. Through the consensus diagnosis process, some of these participants were assigned a Clinical Dementia Rating (CDR)-like dementia severity rating of 0.5 and associated with the diagnosis of cognitive impairment no dementia (CIND). We further evaluated the SCI models by calculating the association between the subset of cases diagnosed as CIND and the SCI model outputs.

Statistical significance was defined using a *p*-value < 0.05. All statistical analyses were performed with code written in-house using Python (https://www.python.org/). We did not perform corrections for multiple comparisons, as our aim was to measure the correlation of each SCI with its corresponding target cognitive domain rather than to draw a general conclusion about the presence or absence of an association between sleep and cognition; that is, the primary focus of the study was to estimate effect sizes rather than statistical hypothesis testing.

## Results

**Figure 1:**
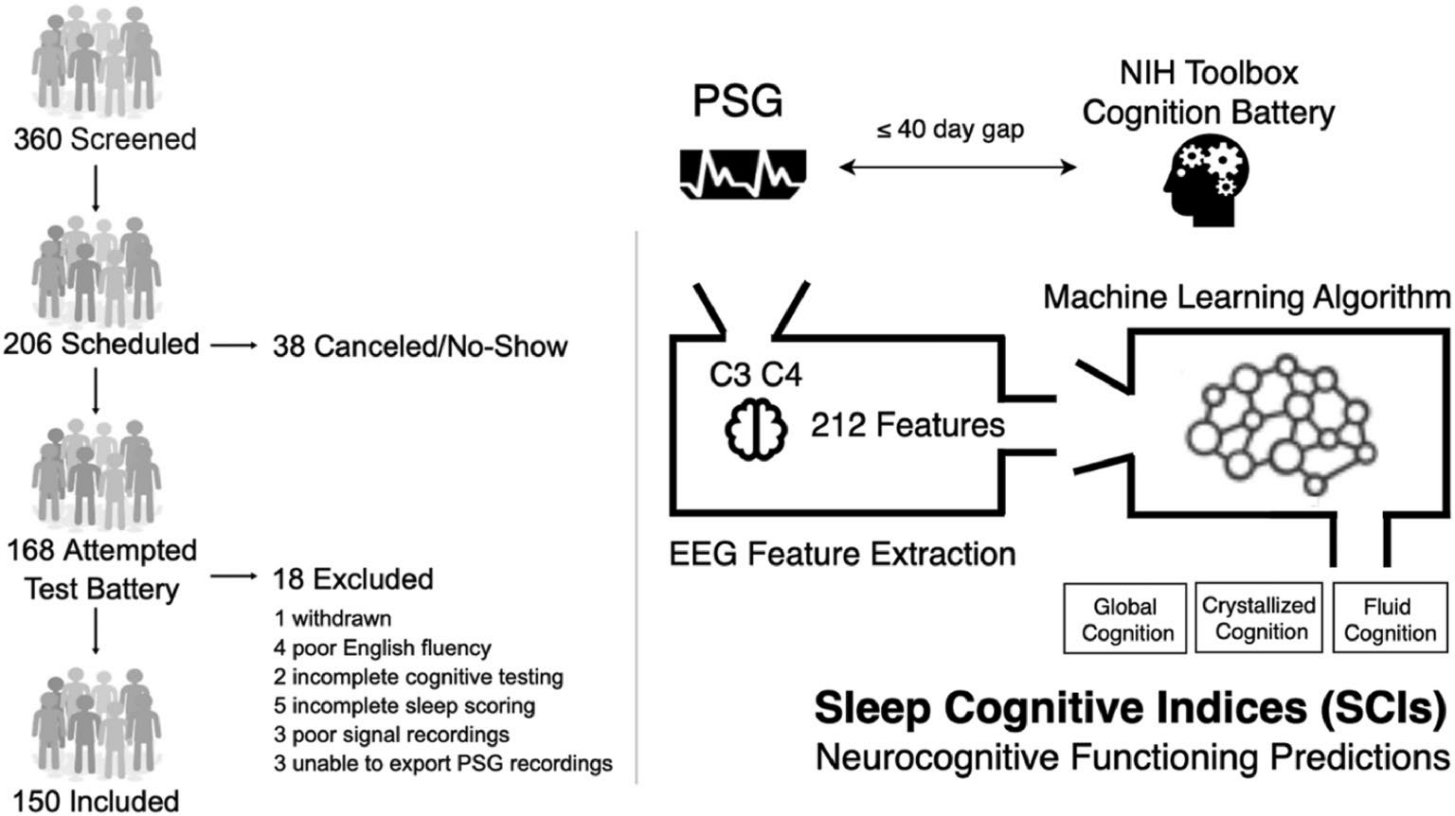
Recruitment flowchart and study design. Flow diagram shows screening and enrollment of study participants, exclusions, and arrival at the final cohort (N = 150).

Overall, 168 participants were enrolled; 18 were subsequently excluded from analysis as they were determined to be ineligible or had missing or incomplete data. A flowchart illustrating the screening and enrollment of study participants is shown in Fig. 1. The final cohort included 150 participants (56% female) with a mean age of 48.8 ± 17.7 years. Participant characteristics are listed in Table 1. The median score for each cognitive test is listed in Table S2.

### Correlations of cognitive scores with sleep cognitive indices

**Figure 2:**
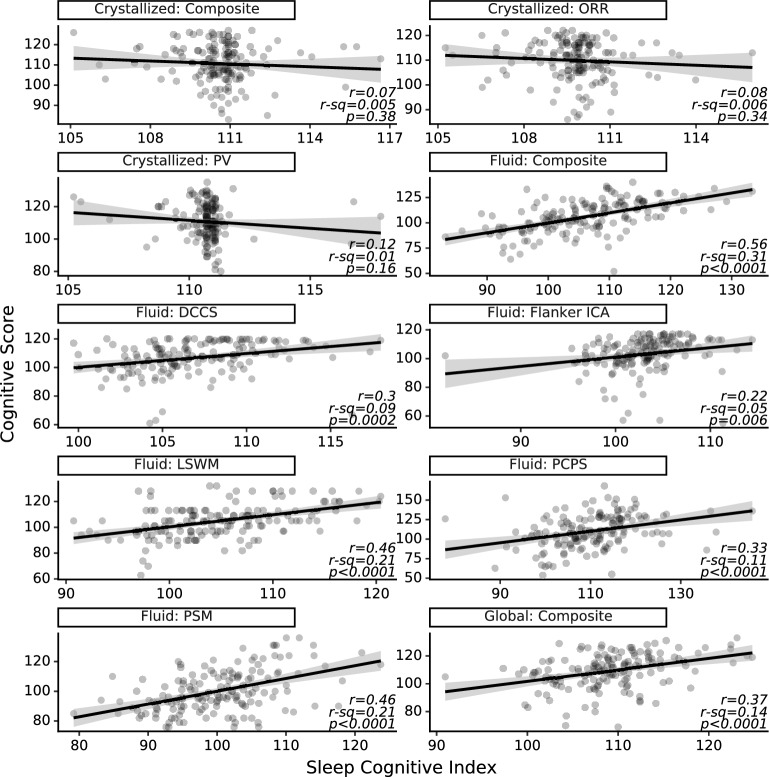
Sleep Cognitive Index is moderately associated with total and fluid cognition and not associated with crystallized cognition. Scatter plots of the absolute (N = 150) and predicted scores for each subtest and composite measures on the NIH Toolbox Cognition Battery are shown below. True cognitive scores are compared with cognitive scores predicted by an Elastic Net model for each cognitive test and composite measure. Sleep spindle features were generated using Luna. *Abbreviations* DCCS, Dimensional change card sort; ICA, Inhibitory control & attention; LSWM, List sorting working memory; ORR, Oral reading recognition; PCPS, Pattern comparison processing speed; PSM, Picture sequence memory; PV, Picture vocabulary.

Figure 2 shows the correlation between SCI and each cognitive test. SCI designed for specific cognitive measures showed significant correlations with total (r = 0.37, *p* < 0.0001) and fluid cognitive scores (r = 0.56, *p* < 0.0001), in addition to each of the five fluid subtests (PCPS: r = 0.33, *p* < 0.0001; Flanker ICA: r = 0.22, *p* = 0.006; LSWM: r = 0.46, *p* < 0.0001; DCCS: r = 0.30, *p* = 0.0002; PSM: r = 0.46, *p* < 0.0001). The SCI designed for measure of crystallized cognition performed poorly (r = − 0.07, *p* = 0.38), as did the SCI designed for its subtests (PV: r = − 0.12, *p* = 0.16; ORR: r = − 0.08, *p* = 0.34). We also show the correlation matrix when using the different SCI models to predict each cognitive score (Fig. S2).

SCI indicators were normally distributed for all significant SCI models (Fig. S3). The top five features for significant SCI models are listed in Table S3. When evaluating the effect of EEG electrode location, we observed similar performance for the three composite cognition SCI models across different subsets of EEG electrodes (Table 3).

**Table 3:** Effect of EEG electrode placement on SCI performance (MAE: mean absolute error).

SCI	Frontal (F3/F4)		Central (C3/C4)		Occipital (O1/O2)		All electrodes	
	MAE	Pearson’s r	MAE	Pearson’s r	MAE	Pearson’s r	MAE	Pearson’s r
Total	4.06	0.34	4.19	0.37	4.39	0.34	4.4	0.34
Fluid	6.61	0.51	7.29	0.56	6.85	0.50	6.69	0.52
Crystallized	0.50	− 0.09	0.90	− 0.07	0.49	− 0.16	0.47	− 0.20

As shown in Table S4, SCI showed stronger correlations than BAI with both total (z = 3.59, *p* = 0.0003) and fluid cognition (z = 4.39, *p* < 0.0001). For fluid subtests, SCI had stronger correlations with LSWM (z = 3.83, *p* = 0.0001), DCCS (z = 2.31, *p* = 0.02), and PSM cognitive tasks (z = 2.53, *p* = 0.01) and similar correlations with the Flanker ICA (z = 1.4, *p* = 0.16) and PCPS tests (z = 1.2, *p* = 0.23). No difference in correlation between SCI and BAI was evident for crystallized composite (z = − 1.59, *p* = 0.11) and subtest scores (PV: z = − 1.16, *p* = 0.25; ORR: z = − 1.23, *p* = 0.22).

**Figure 3:**
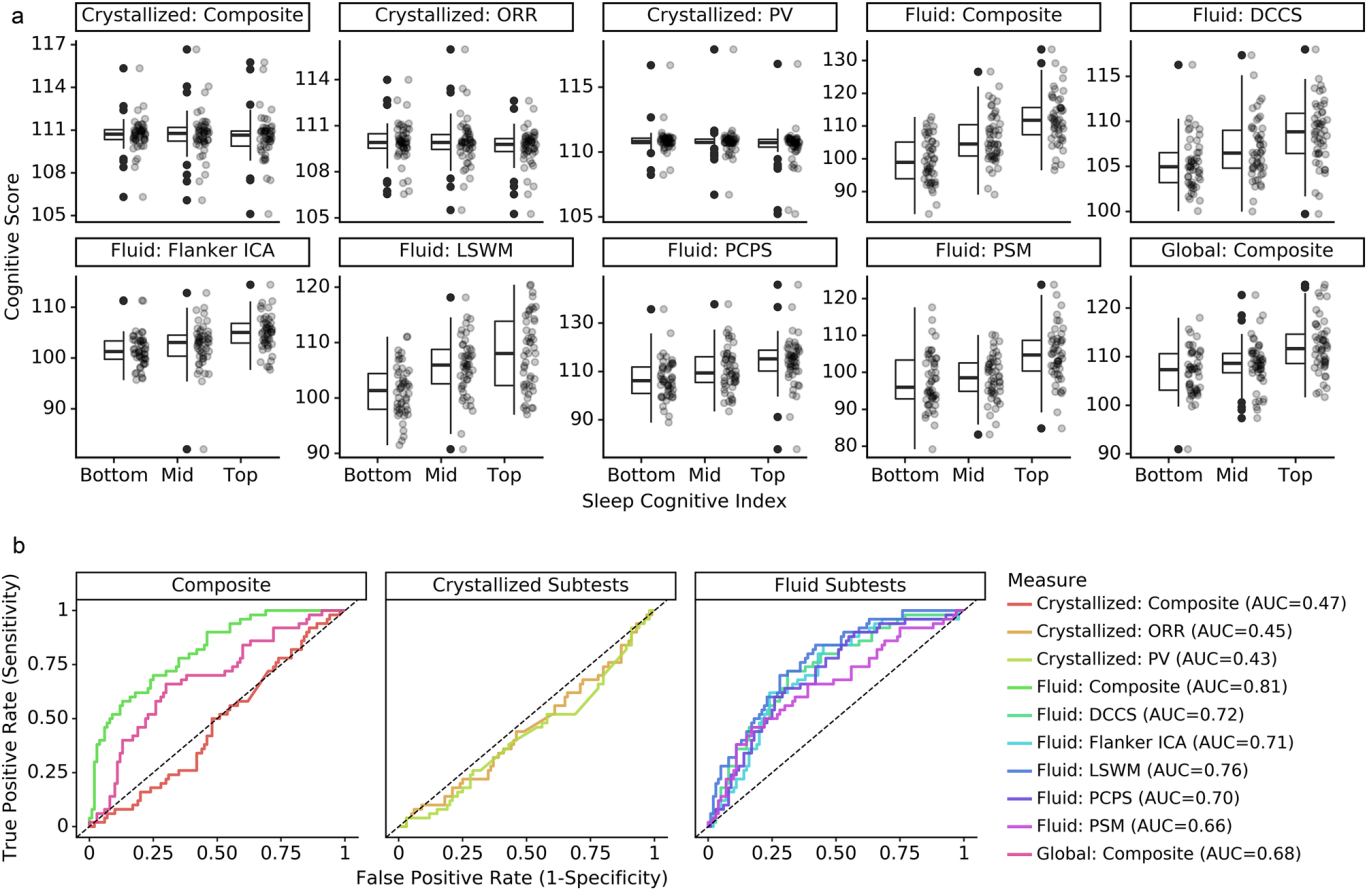
Analysis of discriminability of the Sleep Cognitive Index. (**a**) Sleep Cognitive Index models discriminated between high and low performers at the group level for total and fluid cognition. (**b**) ROC curve for each cognitive test. Cognitive scores (N = 150) were predicted using an Elastic Net model for each cognitive test and composite measure on the NIH Toolbox Cognition Battery. *Abbreviations* DCCS, Dimensional change card sort; ICA, Inhibitory control & attention; LSWM, List sorting working memory; ORR, Oral reading recognition; PCPS, Pattern comparison processing speed; PSM, Picture sequence memory; PV, Picture vocabulary.

To evaluate the ability of SCI indicators to discriminate high versus low cognitive scores at the group level, we conducted Cuzick’s test for trend and found strong trends for total SCI (z = 4.72, *p* < 0.0001), fluid SCI (z = 7.06, *p* < 0.0001), and all fluid subtest SCIs (DCCS: z = 5.21, *p* < 0.0001; Flanker ICA: z = 5.15, *p* < 0.0001; LSWM: t = 5.16, *p* < 0.0001; PSM: t = 4.67, *p* < 0.0001; PCPS: t = 4.61, *p* < 0.0001). The SCI designed for the crystallized cognition score (z = − 0.86, *p* = 0.39) and subtest scores (PV: t = − 1.98, *p* = 0.05; ORR: t = − 1.42, *p* = 0.15) did not display any significant or meaningful trends (Fig. 3a). Receiver Operating Characteristic (ROC) curves and Area Under Curve (AUC) scores confirmed that SCI models could differentiate low versus high scorers at the individual level for fluid and total cognition composite and subset scores (AUC ranged from 0.74 to 0.90), but not for crystallized composite and subset scores (AUC ranged from 0.38 to 0.46). ROC curves are shown in Fig. 3b.

**Figure 4:**
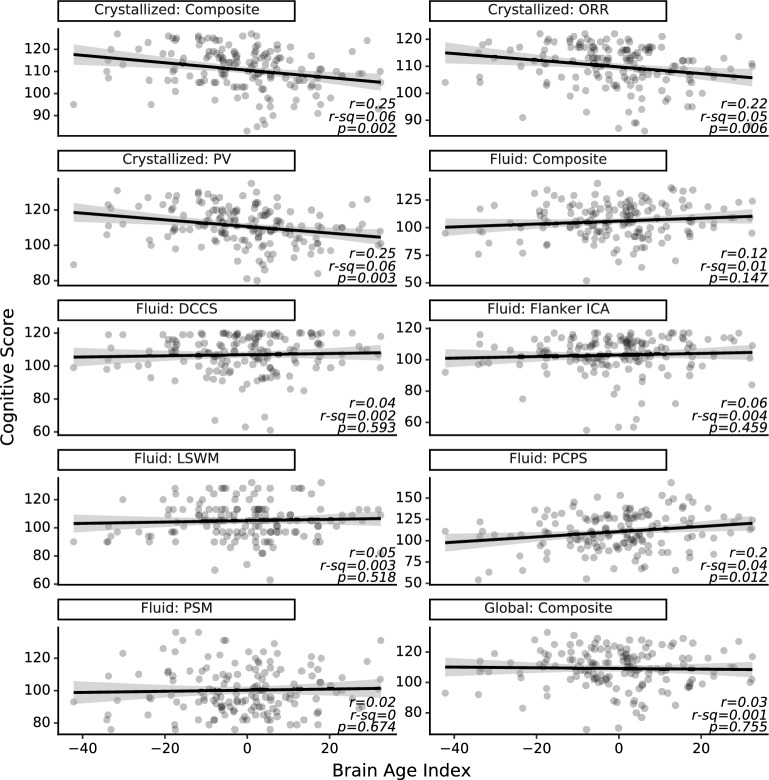
Brain Age Index is moderately associated with crystallized cognition and not associated with total and fluid cognition. Scatter plots of BAI and the absolute scores (N = 150) for each subtest and composite measures on the NIH Toolbox Cognition Battery are shown below. *Abbreviations* DCCS, Dimensional Change Card Sort; ICA, Inhibitory Control & Attention; LSWM, List Sorting Working Memory; ORR, Oral Reading Recognition; PCPS, Pattern Comparison Processing Speed; PSM, Picture Sequence Memory; PV, Picture Vocabulary.

Examination of BAI showed that among the three major cognition domains, only crystallized cognition exhibited a significant correlation, which was negative (Crystallized: r = − 0.25, *p* = 0.002; Fluid: r = 0.12, *p* = 0.15; Total: r = − 0.03, *p* = 0.75). Increased BAI was also negatively correlated with both crystallized cognition subtests (PV: r = − 0.25, *p* = 0.003; ORR: r = − 0.22, *p* = 0.006) and was positively correlated with the processing speed fluid subtest (PCPS: r = 0.20, *p* = 0.01; Flanker ICA: r = 0.06, *p* = 0.46; LSWM: r = 0.05, *p* = 0.52; DCCS: r = 0.04, *p* = 0.59; PSM: r = 0.02, *p* = 0.67). Figure 4 shows a scatter plot and linear fit between BAI and each cognitive test. The opposite signs of the correlations between BAI and PCPS (positive correlation) versus crystallized cognition (negative correlation) scores likely reflect the differential age-related changes observed in fluid and crystallized cognition: fluid cognition tends to decline with age and crystallized cognition likely increases to compensate^24^.

### Evaluating cognitive variation related to age, sex, and education

Likelihood ratio tests confirmed that SCI indicators for the three cognition measures and the Flanker ICA, LSWM, PCPS, and PSM cognitive tasks fit the data similarly to a full “brain health” model that incorporated EEG, age, education, and sex features (*p* = 0.1). Therefore, SCI models adequately capture variation in cognitive performance related to these factors. Detailed metrics for all models are listed in Table S5.

### External validation

**Figure 5:**
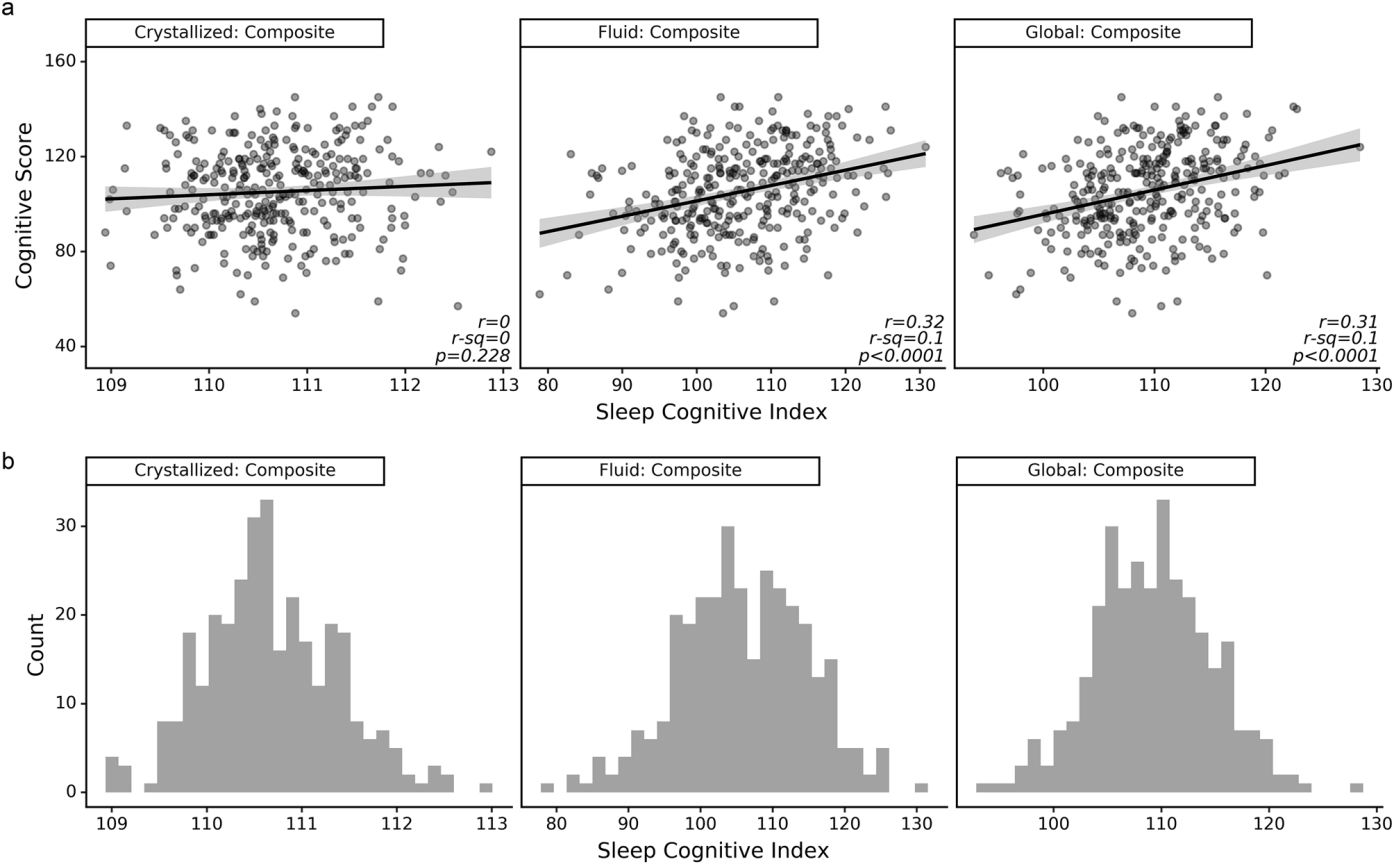
Validation of the three composite cognition SCI models in the Sleep Heart Health Study (SSHS) dataset. (**a**) SCI indicators of fluid and total intelligence are similarly associated with participants’ Wechsler Memory Scale (WMS) scores, while SCI indicators of crystallized intelligence are not associated with WMS scores. (**b**) All three SCI indicators are normally distributed. Participants (N = 324) completed the WMS through the Framingham Heart Study.

Of the 324 SHHS/FHS participants available for analysis, 20 participants had mild cognitive impairment at the time of neuropsychological evaluation. Using all participant data, both total and fluid cognition SCI indicators showed similar correlations with the participants’ total WMS scores (total: r = 0.31, *p* < 0.0001; fluid: r = 0.32, *p* < 0.0001). In contrast, the crystallized cognition SCI model was poorly indicative of participants’ total WMS scores (r = 0.07, *p* = 0.23). Correlations between the three SCI models and cognitive scores, along with score distributions are shown in Fig. 5. No significant change in the strength of association was observed when cognitively impaired participants were excluded from analysis (total: r = 0.30, *p* < 0.0001; fluid: r = 0.30, *p* < 0.0001; crystallized: r = 0.06, *p* = 0.28). Baseline characteristics of patients are listed in Table 4.

**Table 4:** Baseline characteristics of FHS patients (N = 324).

	Female (N = 159)	Male (N = 165)
Age, years, mean ± SD	61.8 ± 1.9	61.8 ± 1.5
Education group		
HS, did not graduate, n (%)	7 (4.4%)	3 (1.8%)
HS graduate, n (%)	53 (33.3%)	53 (32.1%)
Some college, n (%)	43 (27.0%)	48 (29.1%)
College graduate, n (%)	56 (35.2%)	61 (37.0%)
ESS, median [IQR]	6.0 [3.3, 9.0]	7.0 [5.0, 11.0]
AHI, median [IQR]	2.8 [1.0, 8.3]	8.3 [2.8, 14.2]
Normal (< 5), n (%)	100 (62.9%)	73 (44.2%)
Mild sleep apnea (5 ≤ AHI < 15), n (%)	42 (26.4%)	54 (32.7%)
Moderate sleep apnea (15 ≤ AHI < 30), n (%)	14 (8.8%)	26 (15.8%)
Severe sleep apnea (AHI ≥ 30), n (%)	3 (1.9%)	11 (6.7%)
Total Sleep Time, median [IQR]	6.7 [6.0, 7.3]	6.2 [5.7, 6.9]
Sleep Efficiency, median [IQR]	0.9 [0.8, 0.9]	0.9 [0.8, 0.9]
REM Latency, median [IQR]	1.2 [1.0, 1.9]	1.1 [0.8, 1.7]
%N1, median [IQR]	3.9 [2.3, 5.5]	4.6 [3.1, 6.5]
%N2, median [IQR]	52.6 [46.4, 60.0]	60.1 [53.4, 65.4]
%N3, median [IQR]	21.6 [14.7, 29.3]	12.6 [7.8, 19.8]
%REM, median [IQR]	21.1 [17.2, 24.4]	20.5 [16.9, 23.9]
WASO, median [IQR]	47 [27.8, 72.0]	39 [28.0, 68.6]

*Abbreviations* SD, Standard deviation; IQR, Interquartile range; ESS, Epworth sleepiness scale; AHI, Apnea–hypopnea index (# apnea events per hour of sleep) at 4% desaturation for hypopnea.

## Discussion

In this cross-sectional observational study, we demonstrate that machine learning analyses of sleep EEG signals can generate indices that correlate with specific tests of cognition. These novel sleep EEG-derived machine learning models—the SCIs developed in the present study—were optimized to serve as indicators of brain health related to each cognitive task. They achieved a weak to moderate correlation with total cognition, moderate correlation with a composite measure of fluid cognition, and a range of weak to moderate correlations for fluid cognition subtests. SCIs for crystallized cognition and tasks were not correlated with composite crystallized cognition and subtest scores. Crucially, all significant SCI models performed well at differentiating low from high test scorers at the group and individual levels. Overall, our results suggest that overnight sleep EEG is a promising source of indicators of neurocognitive health. This is significant because sleep EEG is increasingly easy to monitor using home devices. Thus, SCIs may have promise for identifying signs of age-related brain diseases that preferentially affect specific aspects of cognitive health and for tracking the physiologic effects of interventions.

### SCI versus BAI

Comparing BAI and SCI performance, we found SCIs exhibited stronger correlations with cognitive scores for total cognition, fluid cognition, and three fluid functional constructs: working memory, episodic memory, and cognitive flexibility. Because fluid cognition often declines at earlier stages of the Alzheimer’s Disease (AD) pathologic cascade^24,37^, measures of fluid cognition may serve as sensitive indicators of preclinical AD and increased vulnerability for cognitive decline in cognitively unimpaired adults.

In contrast, the previously published BAI was correlated with crystallized composite cognition and subtest scores and with the visual processing speed subtest of fluid cognition. No correlation was found between BAI and total cognition, fluid cognition, or the remaining four fluid subtests. Although we anticipated SCI to show stronger associations with cognition, the lack of an association between BAI and fluid cognition was unexpected. On further examination, we found that while crystallized cognition and chronological age (CA) were positively correlated (r = 0.37, *p* = 0.001), BA was negatively correlated with crystallized cognition in our cohort (r = − 0.16, *p* = 0.049). BAI (i.e., BA-CA) was therefore negatively correlated with crystallized cognition. This was not the case for fluid cognition. Because both CA and BA were negatively correlated with fluid cognition, their difference (BAI) was not associated with fluid cognition. The different results for BAI and SCI are in alignment with previous findings that relate distinct brain regions for the two cognition types. When evaluating the effects of different white-matter tracts on fluid and crystallized cognition, one study linked the forceps minor tract with measures of crystallized cognition and the superior longitudinal fasciculus with measures of fluid cognition^23^.

The lack of correlation between SCI and crystallized cognition may have arisen because the features computed did not capture predictive information about crystallized cognition or the choice of model was inadequate for this task due to possible non-linear relationships between sleep features and crystallized cognition.

Including non-EEG metrics of health as features of a cognition index could potentially improve correlations between sleep metrics and cognition. For example, one study that predicted individual sleep metrics using age, cognitive scores, status of cardiometabolic disease, and baseline covariates found that individuals who performed above average within their age group exhibited sleep metrics closer to younger and healthier individuals^38^.

### Most influential features across SCI models

When reviewing the top contributors to significant SCI models, we found that a higher delta-to-theta ratio in N3 was important for total cognition, and a higher delta-to-alpha ratio in N3 was influential for both total cognition and working memory. This finding is in line with previous studies that show decreased delta band power during sleep for older adults with and without sleep disorders^39,40^ and increased delta band power during sleep in response to a motor learning task^41^.

Another N3 feature, line length, significantly contributed to total cognition, fluid cognition, cognitive flexibility, processing speed, and episodic memory models. Line length, also referred to as the mean resultant vector length, is the total variation in the signal amplitude and frequency and is a measure of EEG signal complexity. In our models, a larger signal complexity led to stronger correlations with cognition. This finding was likely driven by the line length of slow oscillations-associated spindles and delta-associated spindles during slow-wave sleep^42^.

Kurtosis of band power was also a strong feature for most models. Kurtosis is a measure of the amount of transiently occurring events, and a larger kurtosis corresponds with a more heavy-tailed distribution. For example, many transient 1-s spindles in a 30-s epoch can lead to higher kurtosis in the sigma band (11–15 Hz). In this study, we found that the tail extremity of alpha band power signals near the onset of sleep and during sleep–wake periods contributed to higher correlations with fluid cognition, working memory, and inhibitory control and visual attention scores. Meanwhile, the tail extremity of theta band power signals during N2 contributed to higher correlations with fluid cognition and four fluid functional constructs, excluding episodic memory. For total cognition, the tail extremity of delta band power signals during REM sleep likewise contributed to higher correlations.

With respect to spindle and slow oscillation features, spindle density during N2 was one of the top three contributors to the composite fluid cognition, inhibitory control and visual attention, cognitive flexibility, and processing speed models. This finding aligns with previous literature that links spindle density with different measures of fluid cognition and functional constructs^43,44^. Spindle amplitude, duration or frequency did not appear to be important. Further, the number of spindles that overlapped with a detected slow oscillation in N2 was an important feature for total cognition and three fluid functional constructs: cognitive flexibility, processing speed, and episodic memory. Coupling between the phase of slow-wave oscillations and spindle activity has been shown to facilitate memory consolidation and performance^45^ and influence cognitive impairment in older adults^46^. These studies further support our episodic memory model, for which the second most influential feature was the circular mean of slow oscillation phase at spindle peak, or mean coupling direction. We also discovered that slow oscillation peak duration predicts cognitive flexibility. Slow oscillation slope, which has been linked to the effectiveness of neuronal synchronization at the cortical level^47^, was also found to predict episodic memory.

Among the sleep architecture features (macrostructure), the percentage of REM sleep was the only highly influential one and ranked the third important feature for working memory. This result agrees with previous studies that support the role of REM duration in working memory performance^48,49^. While REM occurs in the middle of the night, other macrostructures are more likely to be effected by the fact that the MGH dataset is a clinical dataset. For example, for total sleep time (TST), multiple studies have shown a U-shape relationship^50–53^ where overly long or short TST is associated with worse cognition and TST between 6 and 8 h is associated with better cognition. However, the sleep architecture in the sleep lab may not reflect their habitual sleep given that participants are awoken around 6am and experience the “first-night effect” associated with sleep studies. This also affects wake after sleep onset, sleep efficiency, total time in bed, sleep latency and REM latency.

### Goodness of fit

A goodness of fit test showed that SCI models for all cognitive composite scores and all but three subtest scores (picture vocabulary, working memory, and episodic memory) were not improved by adding age and sex features. This suggests that SCI models capture changes in neurocognitive health related to age and sex via features of brain activity during sleep.

### EEG electrodes used in the SCI models

As shown in Table 3, the SCI model trained using central EEG electrodes performs best. This could be explained by the top features: the delta band power during N3 is highest at the central location, therefore the delta-to-theta power ratio during N3 at the central location is the most predictable. Similarly, the spindles at the central electrodes are the so-called fast spindles, which have been shown to correlate with cognition more strongly than slow spindles at frontal electrodes^54^.

### External validation

We investigated whether SCI designed for the three proposed measures of cognition were indicative of performance on the WMS in the SHHS/FHS dataset and found significant correlations between SCI for fluid and total cognition and WMS scores. Both models resulted in comparable correlations, while the crystallized model had no correlation. Compared to our MGH dataset, the overall performance for total and fluid cognition SCI models was reduced in the SHHS/FHS dataset. This difference in performance is likely driven by differences in methodologies, such as the use of different neuropsychological batteries (the WMS selected does not include specific measures of processing speed or working memory), the larger gap between neuropsychological and polysomnography exam dates (SHHS/FHS ≤ 1095 days; MGH ≤ 40 days), and the difference in the average age of the two cohorts (SHHS/FHS: 62 years; MGH: 49 years). Sex was evenly divided for both cohorts, while the level of education could not be compared due to different methods of capturing education levels.

### Limitations

Our study has several limitations, one of which is selection bias. As the study was offered only to those undergoing a PSG for suspected sleep disorders, participants likely had at least subjectively abnormal sleep. In addition, we did not control for medications. Therefore, the cohort would not be reflective of a healthy population. Participants also lacked racial (76% White) and socioeconomic diversity. As the single in-lab PSG setting is known to create the first-night effect, sleep for some participants may not represent typical sleep at home. Lastly, noise in the cognition scores may exist, as we did not control for the time of day when administrating the cognitive test battery or the time between PSG and cognitive assessment.

### Future directions

In future research, the night-to-night variability of SCI should be considered, as our previous work shows that the average night-to-night standard deviation in calculating BAI is 7.5 years, which can be reduced to less than 5 years by averaging consecutive nights^55^. Because calculating SCI only requires two central electrodes, information on night-to-night variability can be conveniently captured using home-based EEG recording devices to improve the reliability of SCI measurements.

Additionally, although BA is commonly measured using magnetic resonance imaging (MRI)^56^, structural MRI scans remain costly, inaccessible to claustrophobic patients and those with metal implants, difficult to deploy or repeat, and do not measure functional status. Thus, sleep EEG-based brain age and health biomarkers may address some of these concerns due to the cost-effectiveness of EEG devices, the accessibility of home-based EEG recording devices, and the aging-associated changes in sleep EEG^57^. To understand the potential benefit in clinical settings, future work is needed to evaluate this biomarker in a more diverse population with cognitive impairment with underlying neuropathologic changes.

## Conclusion

Sleep cognitive indices (SCI) are correlated with measures of total and fluid cognition, while the brain age index (BAI) is correlated with measures of crystallized cognition. Key features contributing to the observed relationships include delta-to-theta and delta-to-alpha band power ratios, kurtosis, spindle density, coupling between slow oscillations and spindles, and percentage of REM sleep. Further research is needed to improve the stability of SCI and to validate SCI as a brain health biomarker.

## Supplementary Information

Supplementary Information.

## Data Availability

The MGH dataset is available from the corresponding author upon reasonable request. The SHHS dataset is available from https://sleepdata.org/datasets/shhs. The cognitive test results from the FHS (overlapping with SHHS) are available from https://www.framinghamheartstudy.org/.

## References

[1] Basak C, Qin S, O'Connell MA (2020). Differential effects of cognitive training modules in healthy aging and mild cognitive impairment: A comprehensive meta-analysis of randomized controlled trials. Psychol. Aging.

[2] LaMantia MA, Stump TE, Messina FC, Miller DK, Callahan CM (2016). Emergency department use among older adults with Dementia. Alzheimer Dis. Assoc. Disord..

[3] Doraiswamy PM, Leon J, Cummings JL, Marin D, Neumann PJ (2002). Prevalence and impact of medical comorbidity in Alzheimer's disease. J. Gerontol. A Biol. Sci. Med. Sci..

[4] Tonelli M (2017). Multimorbidity, dementia and health care in older people: A population-based cohort study. CMAJ Open.

[5] Bradford A, Kunik ME, Schulz P, Williams SP, Singh H (2009). Missed and delayed diagnosis of dementia in primary care: Prevalence and contributing factors. Alzheimer Dis. Assoc. Disord..

[6] Bernstein A (2019). Dementia assessment and management in primary care settings: A survey of current provider practices in the United States. BMC Health Serv. Res..

[7] Ohayon MM, Carskadon MA, Guilleminault C, Vitiello MV (2004). Meta-analysis of quantitative sleep parameters from childhood to old age in healthy individuals: Developing normative sleep values across the human lifespan. Sleep.

[8] Mander BA, Winer JR, Walker MP (2017). Sleep and human aging. Neuron.

[9] Purcell SM (2017). Characterizing sleep spindles in 11,630 individuals from the national sleep research resource. Nat. Commun..

[10] Pace-Schott EF, Spencer RM (2015). Sleep-dependent memory consolidation in healthy aging and mild cognitive impairment. Curr. Top Behav. Neurosci..

[11] Blackwell T (2014). Associations of objectively and subjectively measured sleep quality with subsequent cognitive decline in older community-dwelling men: The MrOS sleep study. Sleep.

[12] Wennberg AMV, Wu MN, Rosenberg PB, Spira AP (2017). Sleep disturbance, cognitive decline, and Dementia: A review. Semin. Neurol..

[13] Winer JR (2021). Association of short and long sleep duration with amyloid-beta burden and cognition in aging. JAMA Neurol..

[14] Sun H (2019). Brain age from the electroencephalogram of sleep. Neurobiol. Aging.

[15] Paixao L (2020). Excess brain age in the sleep electroencephalogram predicts reduced life expectancy. Neurobiol. Aging.

[16] Ye E (2020). Association of sleep electroencephalography-based brain age index with Dementia. JAMA Netw. Open.

[17] Leone MJ (2021). HIV increases sleep-based brain age despite antiretroviral therapy. Sleep.

[18] Buckelmuller J, Landolt HP, Stassen HH, Achermann P (2006). Trait-like individual differences in the human sleep electroencephalogram. Neuroscience.

[19] Berry RB (2015). The AASM Manual for the Scoring of Sleep and Associated Events.

[20] Weintraub S (2014). The cognition battery of the NIH toolbox for assessment of neurological and behavioral function: Validation in an adult sample. J. Int. Neuropsychol. Soc..

[21] Jaeggi SM, Buschkuehl M, Jonides J, Perrig WJ (2008). Improving fluid intelligence with training on working memory. Proc. Natl. Acad. Sci. USA.

[22] Akshoomoff N (2013). VIII. NIH toolbox cognition battery (CB): Composite scores of crystallized, fluid, and overall cognition. Monogr. Soc. Res. Child Dev..

[23] Gongora D (2020). Crystallized and fluid intelligence are predicted by microstructure of specific white-matter tracts. Hum. Brain Mapp..

[24] Li Y, Baldassi M, Johnson EJ, Weber EU (2013). Complementary cognitive capabilities, economic decision making, and aging. Psychol. Aging.

[25] Sun H (2017). Large-scale automated sleep staging. Sleep.

[26] Fisher RA (1915). Frequency distribution of the values of the correlation coefficient in samples from an indefinitely large population. Biometrika.

[27] Redline S (1998). Methods for obtaining and analyzing unattended polysomnography data for a multicenter study. Sleep Heart Health Res. Group Sleep.

[28] Quan SF (1997). The sleep heart health study: Design, rationale, and methods. Sleep.

[29] Zhang GQ (2018). The national sleep research resource: Towards a sleep data commons. J. Am. Med. Inform. Assoc..

[30] Dean DA (2016). Scaling up scientific discovery in sleep medicine: The national sleep research resource. Sleep.

[31] Kannel WB, Feinleib M, McNamara PM, Garrison RJ, Castelli WP (1979). An investigation of coronary heart disease in families. The Framingham offspring study. Am. J. Epidemiol..

[32] Farmer ME (1987). Neuropsychological test performance in framingham: A descriptive study. Psychol. Rep..

[33] Au R, Piers RJ, Devine S (2017). How technology is reshaping cognitive assessment: Lessons from the framingham heart study. Neuropsychology.

[34] McGrath ER (2017). Blood pressure from mid- to late life and risk of incident dementia. Neurology.

[35] Satizabal CL (2016). Incidence of Dementia over three decades in the framingham heart study. N. Engl. J. Med..

[36] Yuan J (2021). Severity distribution of Alzheimer's disease Dementia and mild cognitive impairment in the framingham heart study. J. Alzheimers Dis..

[37] McDonough IM (2016). Discrepancies between fluid and crystallized ability in healthy adults: A behavioral marker of preclinical Alzheimer's disease. Neurobiol. Aging.

[38] Djonlagic I (2021). Macro and micro sleep architecture and cognitive performance in older adults. Nat. Hum. Behav..

[39] Luca G (2015). Age and gender variations of sleep in subjects without sleep disorders. Ann. Med..

[40] Carrier J, Land S, Buysse DJ, Kupfer DJ, Monk TH (2001). The effects of age and gender on sleep EEG power spectral density in the middle years of life (ages 20–60 years old). Psychophysiology.

[41] Huber R, Ghilardi MF, Massimini M, Tononi G (2004). Local sleep and learning. Nature.

[42] Kam K, Pettibone WD, Shim K, Chen RK, Varga AW (2019). Dynamics of sleep spindles and coupling to slow oscillations following motor learning in adult mice. Neurobiol. Learn. Mem..

[43] Ferini-Strambi L, Galbiati A, Marelli S (2013). Sleep microstructure and memory function. Front. Neurol..

[44] Fogel SM, Smith CT (2011). The function of the sleep spindle: A physiological index of intelligence and a mechanism for sleep-dependent memory consolidation. Neurosci. Biobehav. Rev..

[45] Staresina BP (2015). Hierarchical nesting of slow oscillations, spindles and ripples in the human hippocampus during sleep. Nat. Neurosci..

[46] Helfrich RF, Mander BA, Jagust WJ, Knight RT, Walker MP (2018). Old brains come uncoupled in sleep: Slow wave-spindle synchrony, brain atrophy, and forgetting. Neuron.

[47] Riedner BA, Vyazovskiy VV, Huber R (2007). Sleep homeostasis and cortical synchronization: III. A high-density EEG study of sleep slow waves in humans. Sleep.

[48] Lau EY, Wong ML, Lau KN, Hui FW, Tseng CH (2015). Rapid-eye-movement-sleep (REM) associated enhancement of working memory performance after a daytime nap. PLoS ONE.

[49] Reichert CF (2014). The circadian regulation of sleep: Impact of a functional ADA-polymorphism and its association to working memory improvements. PLoS ONE.

[50] Sternberg DA, Ballard K, Hardy JL, Katz B, Doraiswamy PM, Scanlon M (2013). The largest human cognitive performance dataset reveals insights into the effects of lifestyle factors and aging. Front. Hum. Neurosci..

[51] Xu L, Jiang CQ, Lam TH (2011). Short or long sleep duration is associated with memory impairment in older Chinese: The Guangzhou biobank cohort study. Sleep.

[52] Gildner TE, Liebert MA, Kowal P, Chatterji S, Snodgrass JJ (2014). Associations between sleep duration, sleep quality, and cognitive test performance among older adults from six middle income countries: Results from the study on global ageing and adult health (SAGE). J. Clin. Sleep Med..

[53] Devore EE, Grodstein F, Duffy JF, Stampfer MJ, Czeisler CA, Schernhammer ES (2014). Sleep duration in midlife and later life in relation to cognition. J. Am. Geriatr. Soc..

[54] Fernandez LMJ, Lüthi A (2020). Sleep spindles: Mechanisms and functions. Physiol. Rev..

[55] Hogan J (2021). Night-to-night variability of sleep electroencephalography-based brain age measurements. Clin. Neurophysiol..

[56] Cole JH (2018). Brain age predicts mortality. Mol. Psychiatry.

[57] Crowley K, Trinder J, Kim Y, Carrington M, Colrain IM (2002). The effects of normal aging on sleep spindle and K-complex production. Clin. Neurophysiol..

