# Supplementary material for: Decoding information about cognitive health from the brainwaves of sleep
Source: Sci Rep. 2023 Jul 15;13:11448. doi: 10.1038/s41598-023-37128-7 (PMC10349883; doi:10.1038/s41598-023-37128-7)

Supplemental Digital Content

Decoding Information about Cognitive Health from the Brainwaves of Sleep

Noor Adra, BA [a,b,c*], Lisa W. Dümmer, MD [a,b,d*], Luis Paixao, MD, MSc [a,b,e*], Ryan A. Tesh, BSc [a,b,c*], Haoqi Sun, PhD [a,b,c*], Wolfgang Ganglberger, MSc [a,b,f], Mike Westmeijer, MSc [b,e,g], Madalena Da Silva Cardoso, MSc [a,b], Anagha Kumar, BA [a,b], Elissa Ye, MSc [a,b], Jonathan Henry, ALM [a,b,c], Sydney S. Cash, MD, PhD [a,b], Erin Kitchener, PhD [a,b,c], Catherine L. Leveroni, PhD [a], Rhoda Au, PhD [h], Jonathan Rosand, MD, MSc [a,c], Joel Salinas, MD, MSc [h,i], Alice D. Lam, MD, PhD [a,b,c], Robert J. Thomas, MD, MMSc [j **], M. Brandon Westover, MD, PhD [a,b,c **]

Supplemental Methods: Elastic Net regression and nested cross-validation

Supplemental Tables

Table S1: Description of NIH Toolbox Cognition Battery subtests
Table S2: Patients’ scores for the NIH Toolbox Cognition Battery (N=150)
Table S3: Top 5 features for significant Sleep Cognitive Index (SCI) models
Table S4: Summary table for correlations with cognitive tests using SCI and BAI
Table S5: Likelihood ratio test results

Supplemental Figures

Figure S1: Flowchart of 10-fold nested cross-validation for model training
Figure S2: Correlation matrix when using one model to predict another cognitive score
Figure S3: Test scores (N=150) were normally distributed for all Sleep Cognitive Index (SCI) models

Supplemental Methods: Elastic Net regression and nested cross-validation

The goal of Elastic Net regression was to obtain the subset of features that minimize the prediction error for cognitive performance, while simultaneously minimizing the number of features selected. Elastic Net does this by imposing a penalty that combines l1- and l2-norms which promotes sparsity (some coefficients will be exactly zero). The overall strength of penalty "lambda" and the relative strength ratio of l1- and l2 norms "alpha" are denoted as hyperparameters. The inner CV loop was used to obtain the best hyperparameters. We selected lambda from 100 candidate values and alpha from 5 candidate values. For each value of lambda and alpha, we computed the root mean squared error (RMSE) averaged across the inner testing folds of the inner CV loop. The lambda and alpha with the lowest average inner testing RMSE were used. The performance we report is based on the average performance among the 10 testing folds from the outer loop. The selected features we report are based on refitting the regression model using the whole dataset with the median of the lambdas and alphas across the outer CV loops. For detailed steps please refer to Figure S1 in this supplemental material.

Table S1: Description of NIH Toolbox Cognition Battery subtests
NIH Toolbox Cognition (46 – Weintraub; new reference - Gershon RC, Wagster MV, Hendrie HC, Fox NA, Cook KF, Nowinski CJ. NIH toolbox for assessment of neurological and behavioral function. *Neurology*. 2013;80(11 Suppl 3):S2-S6. doi:10.1212/WNL.0b013e3182872e5f; https://www.healthmeasures.net/explore-measurement-systems/nih-toolbox)

Cognitive subdomain	Functional Construct	Composite Cognitive Measure	Toolbox Instrument	Description
Executive function & Attention	Inhibitory control and visual attention	Fluid	Flanker Inhibitory Control & Attention Test Age 12+ v2.1	Ability to sustain and shift attention and inhibit automatic responses that may interfere with achieving goals
Executive function	Cognitive flexibility	Fluid	Dimensional Change Card Sort Test Age 12+ v2.1	Capacity to plan, organize and monitor actions in a goal-oriented manner
Working memory	Working memory for stimuli presented visually and auditorily	Fluid	List Sorting Working Memory Test Age 7+ v2.1	Ability to process and hold information short-term, and manipulate that information
Episodic Memory	Visual episodic memory	Fluid	Picture Sequence Memory Test Age 8+ Form A v2.1	Ability to acquire, store and retrieve new information encoded in a time-specific manner
Processing Speed	Visual processing speed	Fluid	Pattern Comparison Processing Speed Test Age 7+ v2.1	Amount of information that can be processed within a certain time
Language	Vocabulary knowledge & comprehension	Crystallized	Picture Vocabulary Test Age 3+ v2.1	Word knowledge
Language	Reading decoding	Crystallized	Oral Reading Recognition Test Age 3+ v2.1	Oral reading decoding skills (or Ability to read and pronounce words)

Table S2: Patients' scores for the NIH Toolbox Cognition Battery (N=150)

NIH-TB Function Constructs & Composite scores	Median score (IQR)
Inhibitory Control and Attention (Flanker ICA)	104 (99-111)
Cognitive Flexiblity (DCCS)	109 (100-116)
Working Memory (LSWM)	105 (97-113)
Episodic Memory (PSM)	98 (90-110)
Processing Speed (PCPS)	109 (97-126)
Vocabulary (PV)	111 (104-119)
Reading (ORR)	112 (106-116)
Fluid cognition	106 (96-118)
Crystallized cognition	111 (105-118)
Total cognition	110 (102-118)

Table S3: Top 5 features for significant Sleep Cognitive Index (SCI) models (for longer feature name description, please see Table 2) (Crystallized cognition model features are not listed as they are not significant)

Test	Feature	Coefficient Value
Total Cognition	Line length at central channel during N3	1.154
Total Cognition	Kurtosis of delta spectral power at central channel during R	1.035
Total Cognition	COUPL_OVERLAP_N2	1.006
Total Cognition	Delta-to-alpha spectral power ratio at central channel during N3	1.001
Total Cognition	Delta-to-theta spectral power ratio at central channel during N3	0.919
Fluid Cognition	Kurtosis of theta band spectral power at central channel during N2	1.685
Fluid Cognition	DENS_N2	1.639
Fluid Cognition	Line length at central channel during N3	1.431
Fluid Cognition	Kurtosis of alpha band spectral power at central channel during W	-1.231
Fluid Cognition	COUPL_MAG_N3	-1.107
Flanker Inhibitory Control & Attention	Kurtosis of theta band spectral power at central channel during N2	0.419
Flanker Inhibitory Control & Attention	Kurtosis of alpha band spectral power at central channel during W	-0.356
Flanker Inhibitory Control & Attention	DENS_N2	0.353
Flanker Inhibitory Control & Attention	COUPL_MAG_N3	-0.324
Flanker Inhibitory Control & Attention	SYMM2_N3	-0.288
List Sorting Working Memory	Kurtosis of theta band spectral power at central channel during N2	1.948
List Sorting Working Memory	Kurtosis of alpha band spectral power at central channel during W	-1.304
List Sorting Working Memory	Percent of stage R	1.127
List Sorting Working Memory	Delta-to-alpha spectral power ratio at central channel during N3	1.095
List Sorting Working Memory	COUPL_MAG_N3	-0.935

Dimensional Change Card Sort	DENS_N2	0.290
Dimensional Change Card Sort	Kurtosis of theta band spectral power at central channel during N2	0.259
Dimensional Change Card Sort	COUPL_OVERLAP_N2	0.236
Dimensional Change Card Sort	SO_POS_DUR_N2	-0.233
Dimensional Change Card Sort	Line length at central channel during N3	0.231
Pattern Comparison Processing Speed	DENS_N2	1.255
Pattern Comparison Processing Speed	Kurtosis of theta band spectral power at central channel during N2theta_bandpower_kurtosis_C_N2	0.975
Pattern Comparison Processing Speed	Line length at central channel during N3	0.965
Pattern Comparison Processing Speed	Kurtosis of theta band spectral power at central channel during W	0.806
Pattern Comparison Processing Speed	COUPL_OVERLAP_N2	0.728
Picture Sequence Memory	COUPL_OVERLAP_N2	1.282
Picture Sequence Memory	COUPL_ANGLE_N3	-1.068
Picture Sequence Memory	Line length at central channel during N3	1.064
Picture Sequence Memory	SO_SLOPE_POS1_N3	1.002
Picture Sequence Memory	Line length at central channel during N2	0.948

Table S4: Summary table for correlations with cognitive tests using SCI and BAI

Cognition test	Which one has stronger correlation	Correlation with SCI: Pearson's r, p-value	Correlation with BAI: Pearson's r, p-value
Total cognition	SCI > BAI	r = 0.37, p < 0.0001	r = -0.03, p = 0.76
Fluid cognition: composite	SCI > BAI	r = 0.56, p < 0.0001	r = 0.12, p = 0.15
Flanker Inhibitory Control & Attention	n.s.*	r = 0.22, p = 0.006	r = 0.06, p = 0.46
Dimensional Change Card Sort	SCI > BAI	r = 0.30, p = 0.0002	r = 0.04, p = 0.59
List Sorting Working Memory	SCI > BAI	r = 0.46, p < 0.0001	r = 0.05, p = 0.52
Picture Sequence Memory	SCI > BAI	r = 0.46, p < 0.0001	r = 0.02, p = 0.67
Pattern Comparison Processing Speed	n.s.	r = 0.33, p < 0.0001	r = 0.20, p = 0.012
Crystallized cognition: composite	n.s.	r = -0.07, p = 0.38	r = -0.25, p = 0.002
Picture vocabulary	n.s.	r = -0.12, p = 0.16	r = -0.25, p = 0.003
Oral reading recognition	n.s.	r = -0.08, p = 0.34	r = -0.22, p = 0.006

*n.s.: not significant

Table S5: Likelihood ratio test results

Test type[+]	Cognitive Score	Age + Education + Sex + EEG: Log-likelihood*	EEG: Log-likelihood	p-value
	Total	2674.81	2781.09	1
	Crystallized	2846.04	2874.75	1
	Fluid	2621.04	2692.96	1
F	Flanker Inhibitory Control & Attention	2791.24	2900.99	1
F	List Sorting Working Memory	2606.56	2669.36	1
F	Dimensional Change Card Sort	2764.01	2700.65	<0.0001
F	Pattern Comparison Processing Speed	2601.40	2754.77	1
F	Picture Sequence Memory	2640.57	2664.94	1
C	Picture Vocabulary	2774.02	2733.79	<0.0001
C	Oral Reading Recognition	2880.47	2781.65	<0.0001

[+] F: fluid, C: crystallized

* Higher values correspond to a better fit

Figure S1: Flowchart of 10-fold nested cross-validation for model training

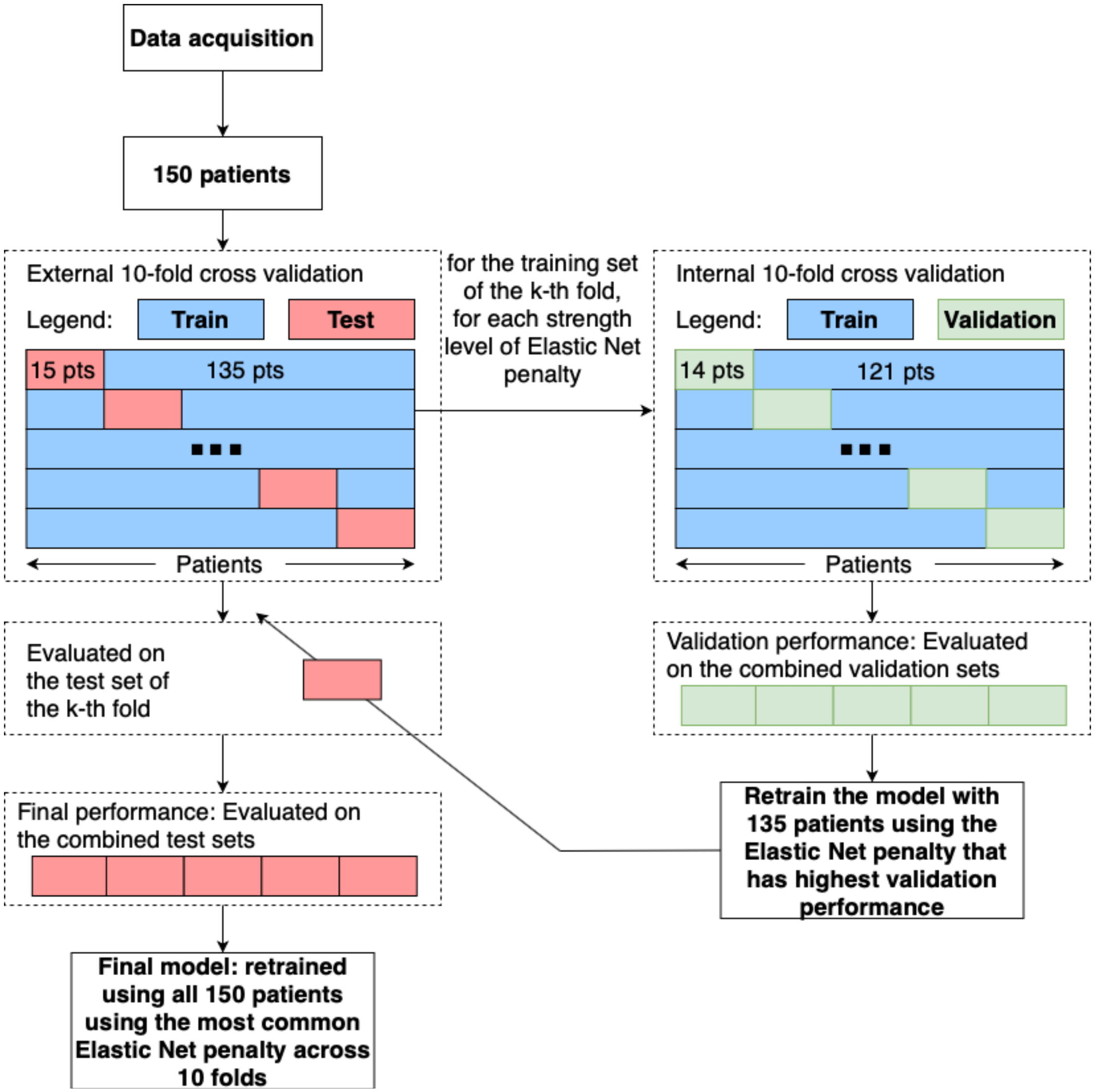

Figure S2: Correlation matrix when using one model to predict another cognitive score. Each cell is the correlation between a model (row, BAI or SCI) and a cognitive score (column). F stands for the fluid intelligence domain. C stands for the crystallized intelligence domain. The color of each grid indicates the amount of correlation (-1 is deep blue and +1 is deep red). Only correlations with p-value < 0.05 is shown with text. BAI: brain age index. Flanker ICA: Flanker inhibitory control and visual attention. LSWM: list sorting working memory. DCCS: dimensional change card sort, measures cognitive flexibility. PCPS: pattern comparison processing speed. PSM: picture sequence memory, measures visual episodic memory. PV: picture vocabulary, measures vocabulary comprehension. ORR: oral reading recognition, measures reading decoding. Composite: composite score from all subtests. "Total: Composite" is the total composite score.

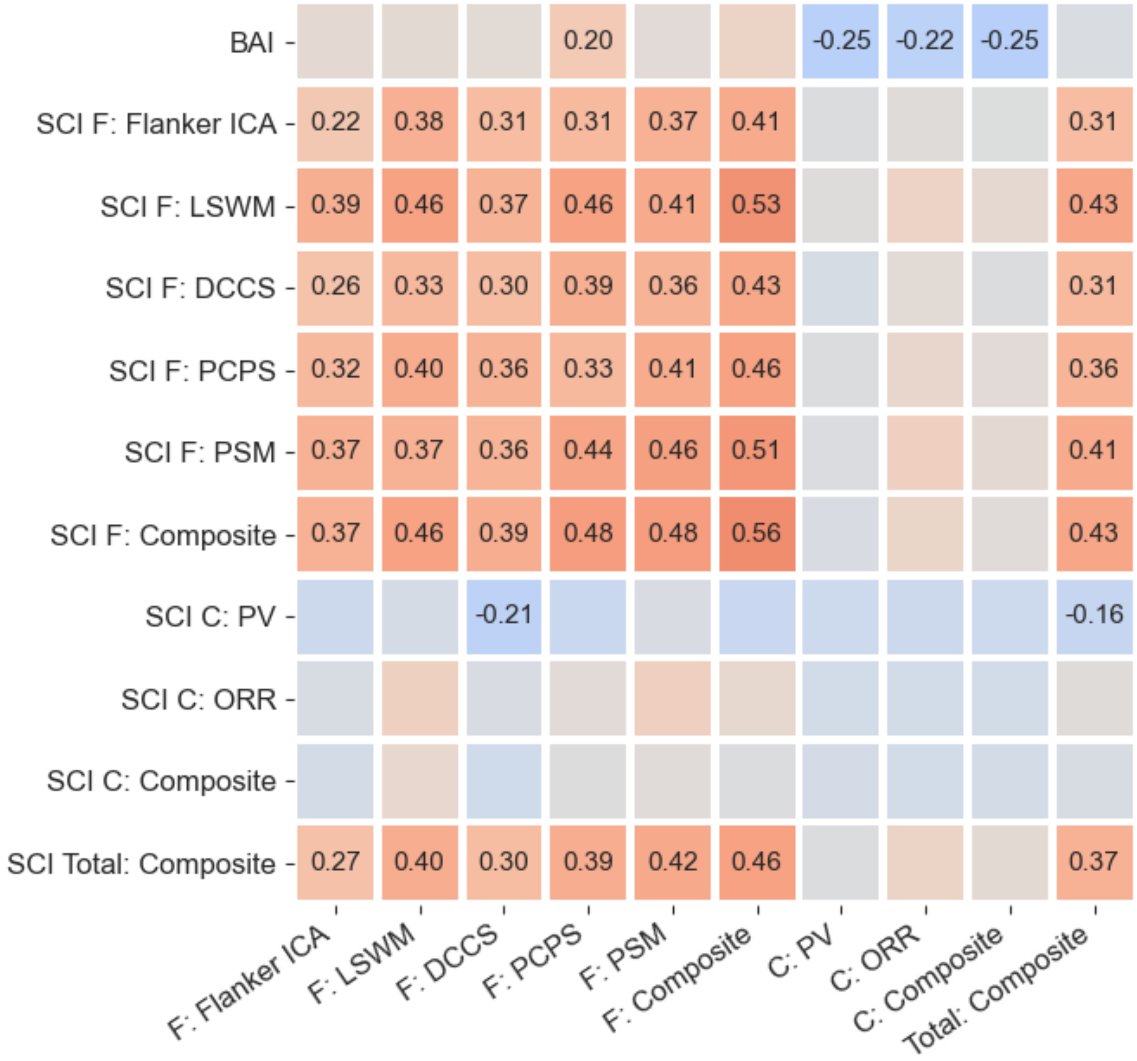

Figure S3: Test scores (N=150) were normally distributed for all Sleep Cognitive Index (SCI) models

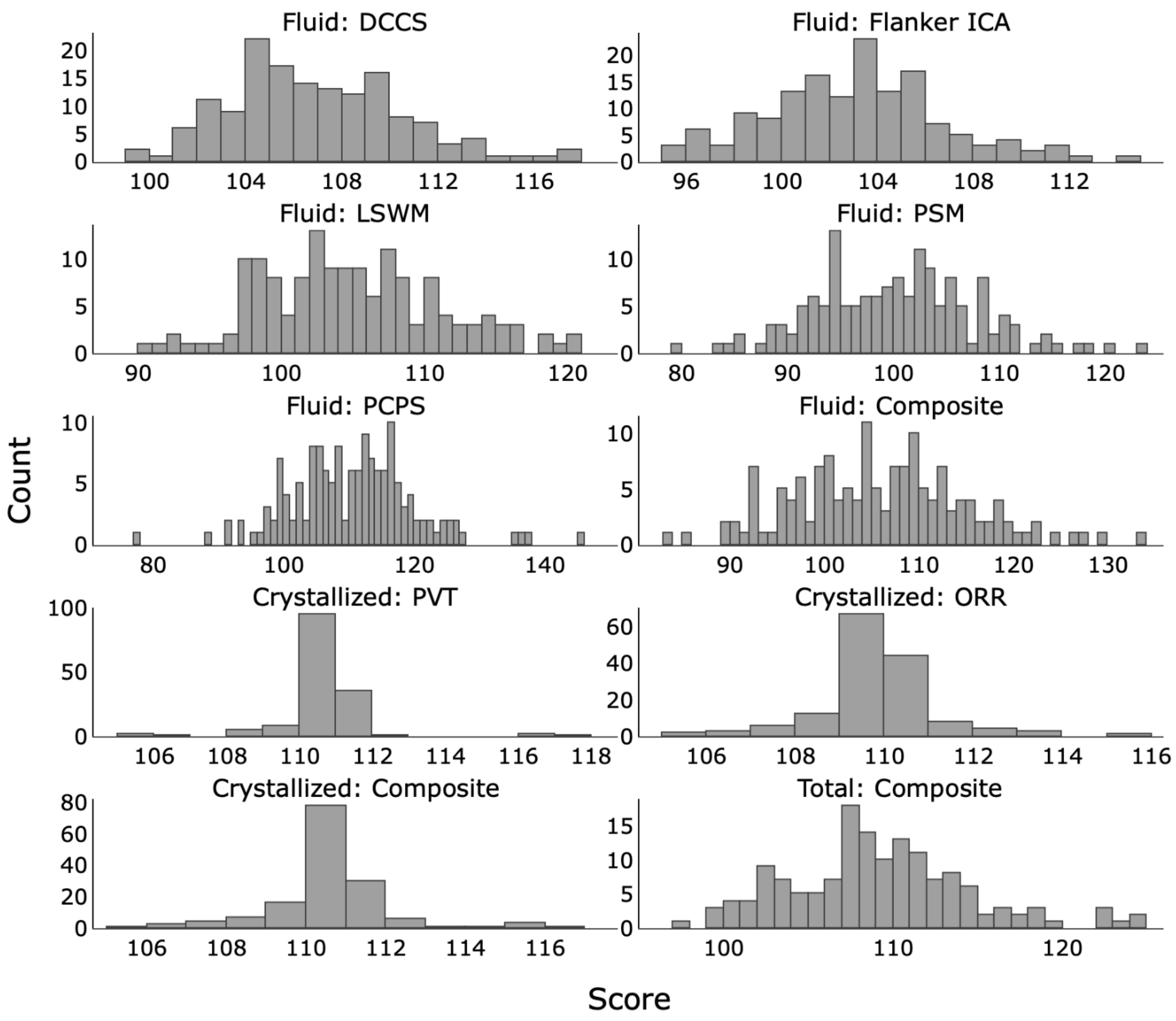

Supplement: Supplementary file 1 — Supplementary Information. [file 41598_2023_37128_MOESM1_ESM.pdf]
